# Bright and water-dispersible membrane probes enable visualization of cellular morphologies and dynamics in light-scattering tissues of living mice†

**DOI:** 10.1039/d5sc03047a

**Published:** 2025-06-20

**Authors:** Takumi Uemura, Ryosuke Kawakami, Hitomi Seki, Satoshi Yoshida, Masamoto Murakami, Takeshi Imamura, Hadano Shingo, Shigeru Watanabe, Yosuke Niko

**Affiliations:** a Research and Education Faculty, Multidisciplinary Science Cluster, Interdisciplinary Science Unit, Kochi University 2-5-1 Akebono-cho, Kochi-shi Kochi 780-8520 Japan y.niko@kochi-u.ac.jp; b Department of Molecular Medicine for Pathogenesism, Graduate School of Medicine, Ehime University Shitsukawa Toon Ehime 791-0295 Japan; c Department of Dermatology, Graduate School of Medicine, Ehime University Shitsukawa Toon Ehime 791-0295 Japan; d Department of Anatomy, Histochemistry and Cell Biology, Miyazaki University 5200 Kihara, Kiyotake Miyazaki 889-1692 Japan; e Department of Geriatric and Environmental Dermatology, Nagoya City University Graduate School of Medical Sciences 1-Kawasumi, Mizuho-cho, Mizuho-ku Nagoya 467-8601 Japan; f Center for Photodynamic Medicine, Kochi Medical School, Kochi University Kohasu, Oko-cho, Nankoku Kochi 783-8505 Japan

## Abstract

Fluorescent plasma membrane probes are indispensable tools for biological studies, enabling the visualization of the fine structure and dynamics of plasma membranes, and, by extension, the overall morphology of living cells. However, their use has been mostly limited to imaging cultured cells or fixed tissue slices. Indeed, few probes have been optimized for visualizing cellular morphologies in intact tissues or organs. Here, we report a new bright squaraine-based membrane probe, dSQ12AQ, which incorporates two anionic anchor groups (sulfonate and long alkyl chain) to ensure high water dispersibility without precipitation—even at concentrations exceeding 10 mg mL^−1^. This highly concentrated probe solution was intravenously administered to living mice without the need for dimethyl solfoxide or other solubilizing agents. Combined with two-photon microscopy, dSQ12AQ enabled clear visualization of whole-cell morphology *in vivo*, allowing dynamic imaging of flowing, rolling, and/or remaining stationary blood cells in the bone marrow vasculature. Moreover, dSQ12AQ extravasated from blood vessels, enabling further staining and visualization of cells in the perivascular bone marrow region. This extravasation was also observed in the hind paw skin, enabling clear visualization of keratinocytes in the epidermis, as well as fibroblasts and eccrine sweat duct cells in the dermis. These results highlight the potential of dSQ12AQ as a valuable tool for *in vivo* studies of various cellular processes and for investigation of refractory or poorly understood diseases and their treatments.

## Abstract 2

Bright membrane probes with high water dispersibility enable *in vivo* visualization of cellular morphology and dynamics in light-scattering tissues.

## Graphical abstract

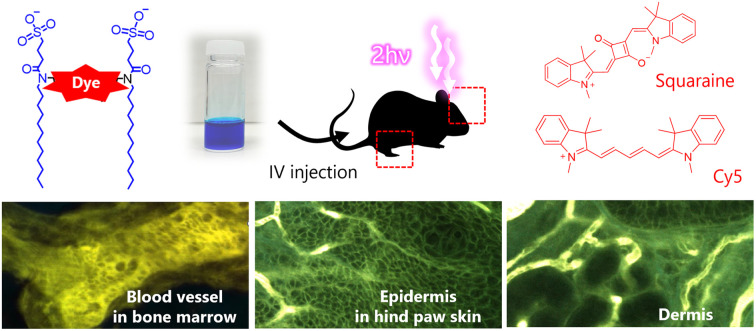

## Introduction

Cell membranes not only separate the intracellular and extracellular environments but also act as a scaffold for fundamental cellular functions, such as signal transduction, membrane transport, and immune responses.^1–6^ Many complex cellular processes, including apoptosis^7^ and metastasis,^8^ involve dynamic changes in cellular morphology, often accompanied or driven by alterations in membrane dynamics. These changes reflect, or even influence, the functional states of cells. Accordingly, techniques for visualizing cell membranes—and thus cellular morphology—are not only critical for detecting and understanding such dynamic behaviors but also provide valuable insights into cellular functions such as the differentiation potential of stem cells, metastatic capacity and drug resistance of cancer cells, and activation status of immune cells.^9^

**Fig. 1:**
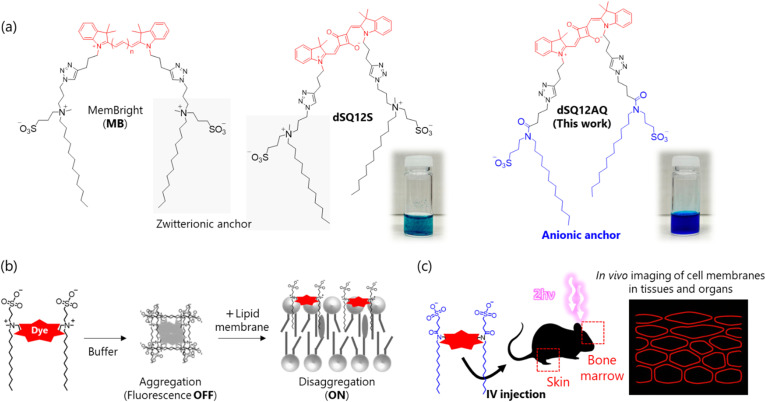
(a) Chemical structures of MB, dSQ12S, and newly developed dSQ12AQ (this study) and (b) their OFF–ON fluorescent behavior in the absence and presence of a lipid membrane. (c) Application of dSQ12AQ for *in vivo* 2PM imaging of cell membranes in living tissues/organs. IV, intravenous; 2PM, two-photon microscopy.

Over the past two decades, various fluorescent probes have been developed to visualize cell membranes in living cells, offering high spatiotemporal resolution and multicolor capability.^10,11^ Among them, the MemBright (MB)^12^ and dSQ12S,^13^ probes developed by Collot *et al.* have gained prominence in biophysics because of their high selectivity for plasma membranes, high one- and two-photon excitation brightness, and photostability (Fig. 1a). These probes, composed of a fluorophore and two anchor groups comprising a long alkyl chain and a zwitterionic moiety, function as fluorogenic amphiphilic molecules, forming non-emissive aggregates (OFF state) in buffer. In the presence of lipid membranes, they bind to the membranes and enter the ON state, exhibiting fluorogenic behavior that is well-suited for wash-free imaging (Fig. 1b). However, their application has been mostly limited to cultured cells rather than live animal tissues and organs, such as those of mice, where wash-free usability is particularly desirable. This limited use is likely related to their poor water dispersibility, as they tend to precipitate in aqueous solutions. As a result, administering highly concentrated probe solutions into live biological environments such as blood vessels can be lethal. In contrast, *in vivo* fluorescence imaging, particularly when targeting light-scattering tissues, typically requires high probe concentrations to achieve sufficient signal intensity.^14,15^ Indeed, probes capable of visualizing the morphology of cell membranes in light-scattering tissues of living animals are expected to become important research tools across a wide range of biological fields, including biomedicine. For instance, among various *in vivo* models and live-cell systems, the skin represents a particularly informative platform, as it exhibits disease-specific morphologies in many refractory skin diseases, such as malignant tumors^16–18^ and inflammatory disorders.^19–23^ Visualizing cell membranes in the skin can therefore aid in detecting such diseases, reveal their pathophysiology, and facilitate the development of effective treatments.

Here, we developed a squaraine-based fluorescent probe for *in vivo* cell membrane imaging, named dSQ12AQ (Fig. 1a). This probe was rationally redesigned to incorporate two anionic anchor groups, enhancing its water dispersibility compared to the dSQ12S and MB probes. A highly concentrated, precipitate-free aqueous solution of dSQ12AQ was successfully prepared and intravenously administered into live mice (Fig. 1c). Using two-photon microscopy (2PM), dSQ12AQ enabled clear visualization of cell morphology *in vivo* by selectively staining cell membranes in light-scattering tissues, including those of the skin, bone marrow, and blood vessels.

## Results and discussion

The synthesis and characterization of dSQ12AQ are described in the ESI (Scheme S1 and Fig. S1–S5).† Briefly, a squaraine dye with two terminal alkynes on the indolenine moieties was synthesized using previously reported procedures.^13^ This molecule was coupled with two anionic anchors bearing an azide group *via* copper(i)-catalyzed azide–alkyne Huisgen cycloaddition to generate dSQ12AQ. This probe was characterized *via* proton nuclear magnetic resonance spectroscopy and high-resolution mass spectrometry. As expected, dSQ12AQ remained well-dispersed at a concentration of 1.0 mg mL^−1^ (*ca.* 0.73 mM) in 20 mM phosphate buffer (PB, pH 7.2) without the aid of dimethyl sulfoxide (DMSO), whereas the model compound dSQ12S rapidly precipitated (see photos shown in Fig. 1a). Remarkably, dSQ12AQ did not precipitate even at a high concentration of 10 mg mL^−1^, which is advantageous for intravenous administration of a large amount of probe.

To confirm whether dSQ12AQ forms aggregates in buffer as illustrated in Fig. 1b, the dependence of its absorption spectra in PB on dye concentration was studied (Fig. S7†). dSQ12AQ exhibited a drastic change in spectral shape between 0.1 and 0.5 μM, and its critical aggregation concentration (CAC) was determined to be around 0.42 μM (Fig. S8†). Dynamic light scattering (DLS) analysis also confirmed the formation of nanosized aggregates of dSQ12AQ (Fig. S9†). Interestingly, dSQ12S did not display a clear CMC at least 0.1–0.5 μM. Considering that the fluorescence intensity of dSQ12S at 0.1 μM is lower than that of dSQ12AQ (Fig. S7†), it is likely that dSQ12S rapidly forms aggregates below 0.1 μM. This difference in CMC between dSQ12AQ and dSQ12S is consistent with their difference in water dispersibility.

**Fig. 2:**
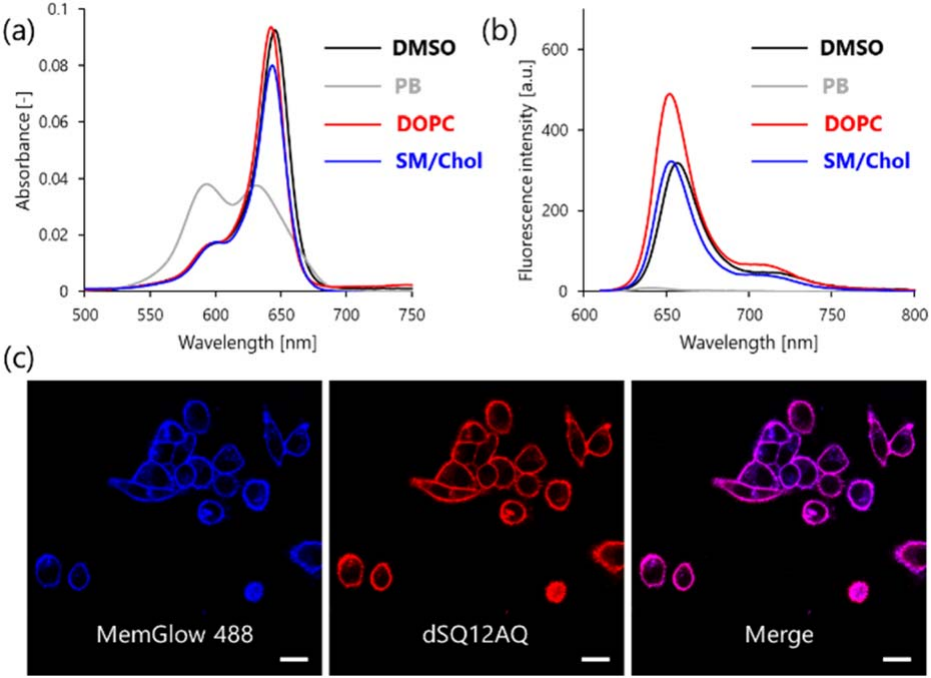
(a) Absorption and (b) fluorescence spectra of dSQ12AQ in DMSO, PB, and in the presence of LUVs. The probe and lipid concentrations were 0.4 and 80 μM, respectively (lipid only for LUVs). (c) Laser scanning confocal microscopy images of unwashed PC-3 cells co-stained with 0.2 μM MB488 and dSQ12A. (Left) Image recorded with the MemGlow 488 (blue) channel (*E*_x_: 473 nm, *E*_m_: 490–525 nm), middle: image recorded with the dSQ12AQ (red) channel (*E*_x_: 635 nm, *E*_m_: 655–755 nm), and (Right) merge of the two channels. Scale bar is 20 μm. Chol, cholesterol; DOPC, 1,2-dioleoyl-*sn*-glycero-3-phosphocholine; LUV, large unilamellar vesicle; SM, sphingomyelin.

Next, the absorption and fluorescence of dSQ12AQ in the absence and presence of lipid membranes were studied (Fig. 2a and b). Detailed spectroscopic data, including molar extinction coefficients and fluorescence quantum yields, are summarized in Fig. S10 and Table S1 of the ESI.† Large unilamellar vesicles (LUVs) with various lipid compositions were used as model membranes.^24^ In the absence of LUVs, dSQ12AQ exhibited a maximum absorption at approximately 590 nm, indicative of the formation of non-emissive H-aggregation.^25,26^ Consistently, no pronounced fluorescence was observed. In contrast, in the presence of LUVs composed of 1,2-dioleoyl-*sn*-glycero-3-phosphocholine (DOPC), which forms a loosely-packed liquid-disordered phase with high fluidity,^24,27^dSQ12AQ showed stronger absorption and fluorescence at approximately 642 and 652 nm, respectively, and the spectral shape observed here was almost identical to that in DMSO, where dSQ12AQ exists in a molecularly dispersed state. This result indicates that dSQ12AQ binds to lipid membranes while dissociating its aggregates. Similar spectral behavior was observed with LUVs composed of sphingomyelin and cholesterol (SM/Chol), which form a tightly packed liquid-order phase with lower fluidity.^24,27^ These results suggest that dSQ12AQ neither precipitated readily nor fully dissolved in buffer, yet maintained fluorogenic behavior comparable to that of dSQ12S.^13^ In fact, it exhibits slightly higher fluorescence quantum yields in LUVs than dSQ12S, likely due to the suppression of intermolecular aggregation on membranes, facilitated by its anionic moieties (Table S1†). The combination of enhanced water-dispersibility and preserved fluorogenic response makes dSQ12AQ particularly suitable for *in vivo* applications, which typically require high probe concentration and are often incompatible with washing steps.^14,15^

To confirm that dSQ12AQ works as a membrane probe, we performed confocal microscopy imaging of cultured cells (human prostate cancer cell line, PC-3) co-stained with a commercially available plasma membrane probe (MemGlow 488)^11^ and dSQ12AQ. Merged images of the blue (MemGlow 488) and red (dSQ12AQ) channels showed strong colocalization, with a Pearson's correlation coefficient of 0.92 (Fig. 2c), indicating that dSQ12AQ can function as a membrane probe. Prior to *in vivo* imaging, the utility of dSQ12AQ for imaging cell membranes in living cultured cells was further explored using advanced confocal microscopy (Fig. S11†). Even at a low concentration of 10 nM, dSQ12AQ clearly visualized cell membranes with a sufficiently high signal-to-noise ratio (SNR), achieving values of 142 ± 38 (mean ± SD), comparable to those obtained with dSQ12S (212 ± 41). Interestingly, despite the higher fluorescence quantum yields of dSQ12AQ in model membranes, its observed SNR was somewhat lower than dSQ12S. This is likely due to the greater hydrophobic character of dSQ12S, which may promote more efficient membrane binding (increasing signal) while facilitating self-quenching of unbound probe in the extracellular space (reducing background). Thus, while dSQ12S may offer slightly better performance for *in vitro* imaging, dSQ12AQ still demonstrates sufficient utility for practical use.

**Fig. 3:**
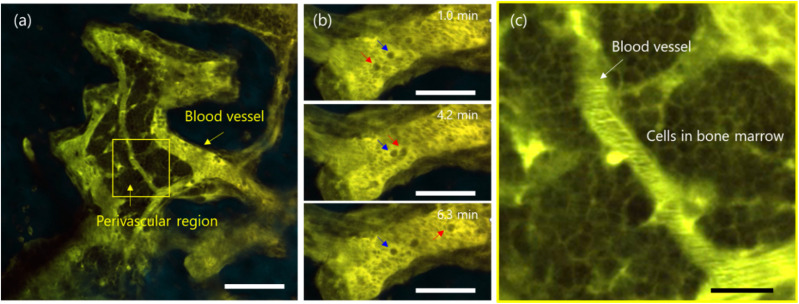
(a) 2PM images of the skull bone marrow in a living mouse, acquired 5 h post intravenous injection of dSQ12AQ (100 μL, 10 mg mL^−1^). (b) Image of the bone marrow vasculature. Red and blue arrows indicate slowly moving and stationary cells, respectively. (c) Image of the perivascular region in bone marrow. *E*_x_: 1100 nm, *E*_m_: 500–550 nm (cyan, second-harmonic generation (SHG)), 560–685 nm (green, fluorescence). Scale bars in (a), (b), and (c) are 100, 50, and 20 μm, respectively. 2PM, two-photon microscopy.

Next, dSQ12AQ was used for *in vivo* 2PM imaging (Fig. 3). A highly concentrated dSQ12AQ buffer solution (100 μL, 10 mg mL^−1^) was intravenously administered to a live mouse, and 2PM imaging was performed 5 h post-injection using an 1100 nm excitation laser. Squaraine dyes can be excited at such long wavelengths, which reduces excitation light scattering and absorption in biological tissues, thereby enhancing the imaging depth.^15,28–30^ Leveraging this property, we first conducted 2PM imaging of the bone marrow of a mouse skull, without the need for surgical procedures such as bone thinning. Bone marrow is a highly scattering tissue and is challenging target in*vivo* imaging, especially when aiming for high spatiotemporal resolution with minimal invasiveness.^31^ Remarkably, our imaging revealed a net-like structure within the bone marrow vasculature (Fig. 3a, b and Movie S1†). These structures were interpreted as individual blood cells, whose plasma membranes were clearly highlighted by dSQ12AQ staining. Although dSQ12AQ appeared to localize slightly to intracellular membranes, likely due to interactions with blood proteins that promote internalization of membrane probes, it still effectively delineated overall cell morphologies. This observation contrasted markedly with conventional blood vasculature imaging using standard fluorescent probes, which typically only delineate the vascular lumen.^14^ Moreover, time-lapse imaging revealed that most of the observed smaller cells flowed rapidly, whereas some larger cells moved slowly along the vessels or even remained stationary (red and blue arrows in Fig. 2b and Movie S2†). The former can be characterized as red blood cells, whereas the latter are likely white blood cells. Interestingly, we did not find dSQ12AQ in the urine of mice, suggesting that most probes bind to blood cell membranes and thus avoid renal excretion, which typically occurs for small molecules. Nevertheless, we observed the cells in perivascular bone marrow, indicating that the probe extravasated from the blood vessels—a phenomenon also typically associated with small molecules (Fig. 3c). This is probably due to the moderate affinity of dSQ12AQ for lipid membranes, which allows it to associate transiently with both blood cells and vascular endothelial cells. Such membrane interaction may facilitate its extravasation into surrounding tissues. These findings demonstrate that the probe is a powerful tool for labeling cell membranes in living tissues and organs, enabling visualization of both cellular morphology and dynamics. Accordingly, it holds significant value for investigating diverse cellular behaviors, including rolling, adhesion, migration, intravasation, and extravasation.

**Fig. 4:**
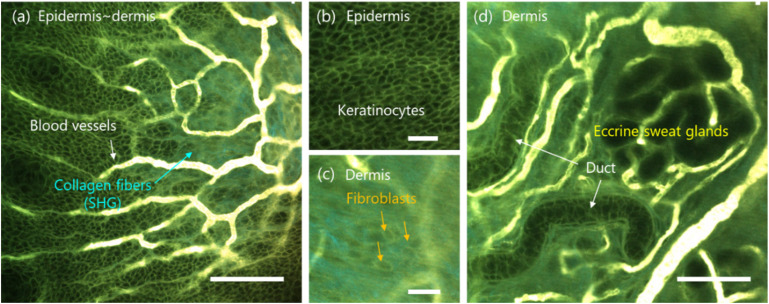
2PM images of the skin tissue of a living mouse, acquired 5 h post-intravenous injection of dSQ12AQ (100 μL, 10 mg mL^−1^). (a) Overview of the tissue structure covering from the epidermis to the dermis. Images of (b) keratinocytes and (c) fibroblasts in the epidermis and dermis region, respectively. (d) Image of the eccrine sweat glands in the dermis region. *E*_x_: 1100 nm, *E*_m_: 500–550 nm (cyan, SHG), 560–685 nm (green, fluorescence). 2PM, two-photon microscopy. SHG, second-harmonic generation.

Next, the skin tissues of live mice were imaged using 2PM. Hind paw skin is considered an important model for human skin due to its relatively thick layers and the presence of eccrine sweat glands.^32^ Nevertheless, conventional 2PM imaging of hind paw skin has been limited, likely due to the lack of a suitable probe capable of staining and imaging such thick tissue.^33^ Based on the favorable results in bone marrow, namely, the extravasation from blood vessels and high brightness under 1100 nm excitation, dSQ12AQ shows strong potential as a probe for skin tissue imaging. Three-dimensional 2PM imaging of skin tissues from the epidermis to the dermis was performed 5 h post-intravenous injection (Fig. 4 and Movie S3†). Remarkably, cells in both the epidermal and dermal region were clearly visualized. This result indicates that dSQ12AQ extravasated from the dermal vasculature, stained cell membranes in the dermis, and subsequently diffused into the epidermis across the basal membrane (Fig. 4a). In the epidermal region (Fig. 4b), dSQ12AQ primarily stained the cell membranes of keratinocytes, making it a potentially valuable tool for detecting skin diseases characterized by distinct cellular morphologies such as malignant tumors including Extramammary Paget's disease^16^ and basal cell carcinoma,^17,18^ as well as inflammatory disorders such as psoriasis vulgaris^20^ and palmoplantar pustulosis.^21–23^ The fibroblast morphology was imaged in the dermal regions, which may be beneficial for studying their role in tissue repair (Fig. 4c). Moreover, dSQ12AQ visualized the eccrine sweat gland duct cells morphology (Fig. 4d). This feature of dSQ12AQ is useful for evaluating the pathophysiology of acquired idiopathic generalized anhidrosis,^34,35^ which significantly diminishes patients' quality of life but remains poorly understood because methods for observing cells in living tissues are lacking.

Finally, all aforementioned *in vivo* 2PM imaging is challenging with the commercial MB series and dSQ12S, as these probes rapidly precipitate in buffer (see the Note in Section 2-4 of the ESI†). The difference between dSQ12S and dSQ12AQ lies in the anchor group, which is zwitterionic or anionic. To validate the utility of the anionic anchor, we synthesized Cy5-12AQ, a water-dispersible analog of MemGlow 640 (member of the MB family),^25^ in which two anionic anchor groups were used rather than zwitterionic anchor groups (see Scheme S1 and Fig. S6†). We found that Cy5-12AQ could also be intravenously administered to mice, enabling its extravasation from blood vessels and subsequent visualization of cell morphologies and dynamics in the hind paw skin (Fig. S12†). These results indicate that introducing anionic anchor groups enhances the water dispersibility of the fluorophore, even if the fluorophore itself is cationic (MB series) or zwitterionic (squaraine) and provides plasma membrane-selectivity and fluorogenic function to the fluorophores. This is an important finding of this work, as plasma membrane probes bearing anionic anchors already exist but their potential for *in vivo* imaging has not been explored.^36,37^ Very recently, a new series of MB probes developed by Danylchuk, Klymchenko, and colleagues has emerged as a promising candidate for *in vivo* imaging applications due to its incorporation of distinct anionic anchors.^38^ However, the effects of fluorophore charges—namely, carbocyanine in the MB series and squaraine in the SQ series—as well as the structure of the anionic anchor on staining behavior and localization remain unclear. Although we have qualitatively observed differences between dSQ12AQ and Cy5-12AQ, particularly in epidermal staining patterns, a comprehensive understanding is lacking. We are therefore currently investigating the detailed staining characteristics of dSQ12AQ across various tissues and organs.

## Conclusion

We developed a squaraine-based cell membrane probe, dSQ12AQ, which exhibits exceptionally high water dispersibility, due to the incorporation of two anionic anchors. This probe remained stably dispersed even at 10 mg mL^−1^ in buffer without precipitation, enabling intravenous administration of the probe at high concentrations to live mice. As the squaraine dye is efficiently excited by a tissue-penetrative 1100 nm laser, high-quality 2PM imaging was achieved in the bone marrow and hind paw skin tissue of live mice. 2PM imaging revealed that dSQ12AQ primary stained the plasma membranes of blood cells, allowing for visualization of their morphologies and dynamics. Furthermore, the probe extravasated from the blood vessels, diffused, and stained bone marrow cells. Similar extravasation and staining behavior were observed in the hind paw skin tissue, where the morphology and dynamics of keratinocytes in the epidermis, fibroblasts, and cells in the duct portion of the eccrine sweat glands were visualized. These features of dSQ12AQ were also observed in carbocyanine dye with two anionic anchors, highlighting the general importance of anchor design for achieving high water dispersibility, membrane targeting, and fluorogenic performance. The new probes presented here will be beneficial for studying various cellular processes and for examining the pathophysiology of refractory and/or poorly understood diseases including malignant tumors, inflammatory disorders, and acquired idiopathic generalized anhidrosis.

## Author contributions

TU conducted molecular synthesis, LUV preparation, and spectroscopic measurements and wrote the manuscript. RK conducted *in vivo* 2PM imaging and image analysis. HS conducted *in vitro* imaging and image analysis. SY and MM contributed to characterization of the skin tissue structure and writing the manuscript. TI supported *in vivo* 2PM imaging, data analysis, and review of the manuscript. SH and SW helped synthesize the molecules, performed spectroscopic analysis, and reviewed the manuscript. YN handled conceptualization, writing, and editing of the manuscript.

## Conflicts of interest

There are no conflicts to declare.

## Supplementary Material

SC-OLF-D5SC03047A-s001

SC-OLF-D5SC03047A-s002

SC-OLF-D5SC03047A-s003

SC-OLF-D5SC03047A-s004

## Data Availability

Experimental data are available from the corresponding author upon reasonable request.
