# Supplementary material for: Bright and water-dispersible membrane probes enable visualization of cellular morphologies and dynamics in light-scattering tissues of living mice
Source: Chem Sci. 2025 Jun 20;16(29):13368–73. doi: 10.1039/d5sc03047a (PMC12188652; doi:10.1039/d5sc03047a)
Supplement: SC-016-D5SC03047A-s004 [file SC-016-D5SC03047A-s004.pdf]

Supplementary Information (SI) for Chemical Science.
This journal is © The Royal Society of Chemistry 2025

Bright and Water-Dispersible Membrane Probes Enable Visualization of Cellular Morphologies and Dynamics in Light-Scattering Tissues of Living Mice

Takumi Uemura,[†,a] Ryosuke Kawakami,[†,b] Hitomi Seki,[a] Satoshi Yoshida,[c] Masamoto Murakami,[d,e] Takeshi Imamura,[b] Hadano Shingo,[a] Shigeru Watanabe,[a] Yosuke Niko*[,a,f]

a. Research and Education Faculty, Multidisciplinary Science Cluster, Interdisciplinary Science Unit, Kochi University, 2-5-1 Akebono-cho, Kochi-shi, Kochi 780-8520, Japan.
b. Department of Molecular Medicine for Pathogenesism, Graduate School of Medicine, Ehime University, Shitsukawa, Toon, Ehime 791-0295, Japan.
c. Department of Dermatology, Graduate School of Medicine, Ehime University, Shitsukawa, Toon, Ehime 791-0295, Japan
d. Department of Anatomy, Histochemistry and Cell Biology, Miyazaki University, 5200 Kihara, Kiyotake, Miyazaki 889-1692, Japan.
e. Department of Geriatric and Environmental Dermatology, Nagoya City University Graduate School of Medical Sciences, 1-Kawasumi, Mizuho-cho, Mizuho-ku, Nagoya 467-8601, Japan.
f. Center for Photodynamic Medicine, Kochi Medical School, Kochi University, Kohasu, Oko-cho, Nankoku, Kochi 783-8505, Japan.

*Corresponding author. E-mail: y.niko@kochi-u.ac.jp
†These authors contributed equally to this work.

Table of Contents

1. **Materials**……………………………………………………….……………………… S3

   1-1. Reagents.

   1-2. Preparation of large unilamellar vesicles (LUVs)

   1-3. Preparation of human prostate cancer cell line (PC-3) cells

   1-4. Animals

2. **Methods**……………………………………………………………………………... S4

   2-1. Characterization of materials

   2-2. Measurements of the photophysical properties

   2-3. Cellular imaging using confocal laser scanning fluorescence microscopy

   2-4. Determination of critical aggregation concentration

   2-4. *In vivo* imaging using two-photon microscopy (2PM)

3. **Synthesis of dSQ12AQ and Cy5-12AQ**………………………………………………… S6

4. **Aggregation behavior of dSQ12AQ and dSQ12S in PB**…………………………… S12

5. **Spectroscopic properties of dSQ12AQ and dSQ12S**…………………………..…….. S14

6. ***In vitro* imaging comparison between dSQ12AQ and dSQ12S**……………….. S15

7. ***In vivo* 2PM imaging using Cy5-12AQ**..S17

8. **Supplemental Movies**………...S18

9. **References**..S19

1. Materials

1-1. Reagents: Unless otherwise noted, all reagents and chemicals were used without further purification. 4-Bromobutanoic acid tert-butyl ester, *N*-ethyldiisopropylamine (DIPEA), trifluoromethanesulfonic anhydride, 1,2-dioleoyl-sn-glycero-3-phosphocholine (DOPC), and cholesterol (Chol) were purchased from Tokyo Chemical Industry Co., Ltd. (Tokyo, Japan). *N*,*N*-Dimethylformamide (DMF), methanol (MeOH), ethanol (EtOH), 1-butanol, 1,2-dichloroethane, ethyl acetate (EtOAc), sodium iodide, sodium azide, trifluoroacetic acid (TFA), pyridine, sodium L-ascorbate, copper(II) sulfate pentahydrate, phosphate buffer (PB buffer, 0.1 mM, pH 7.2), and anhydrous magnesium sulfate ($MgSO_4$) and sodium sulfate (Na_2SO_4) were obtained from Nacalai Tesque, Inc. (Kyoto, Japan). Chloroform, dichloromethane (DCM), and hexane were purchased from Kishida Chemical Co., Ltd. (Osaka, Japan). Bovine brain sphingomyelin (SM) and (1-cyano-2-ethoxy-2-oxoethylidenaminooxy)dimethylaminomorpholinocarbenium hexafluorophosphate (COMU) were obtained from Merck KGaA (Darmstadt, Germany). Anhydrous DCM and DMF were purchased from FUJIFILM Wako Pure Chemical Corporation (Osaka, Japan). Squaraine and Cy5 derivatives bearing two terminal alkyne groups, referred to as compound **5**[1] and **6**[2], respectively, were synthesized as described in the literatures. 3-(dodecylammonio)propane-1-sulfonate was prepared according to a literature[3].

1-2. Preparation of large unilamellar vesicles (LUVs): LUVs were obtained by the extrusion method as previously described.[4] Briefly, a suspension of multilamellar vesicles was extruded using a Mini-Extruder (Avanti Polar Lipids, Inc., AL, USA). The pore size of the filter was 100 nm (10 passages), which generated monodisperse LUVs with a mean diameter of 100 nm, as measured with a dynamic light scattering (DLS) system (ELSZneo (638 nm) or NanoSAQLA (660 nm), Otsuka Electronics Co. Ltd., Osaka, Japan) based on the CONTIN method.

1-3. Preparation of human prostate cancer cell line (PC-3) cells: The PC-3 cells were maintained in Dulbecco's modified Eagle medium (DMEM) supplemented with 10% (v/v) fetal bovine serum (FBS; HyClone, Thermo Fisher Scientific, Waltham, MA, USA) and a 1% (v/v) antibiotic antimycotic solution (09366-44, Nacalai Tesque, Kyoto, Japan). The cells were stored under 5% CO_2 gas at 37 °C.

1-4. Animals: Adult (8-week-old) C57BL/6 mice were used for in vivo imaging. All animals were housed under a 12:12-hour light-dark cycle (lights on from 08:00 to 20:00) with ad libitum access to food and water, and in an environment with controlled temperature (23 ± 2 °C) and humidity (over 30%). All animal experiments were approved by the Ethics Committee for Animal Experiments of Ehime University (#05RE2–16), and all procedures were conducted in accordance with the approved guidelines.

2. Methods

2-1. Characterization of materials: ^{1}H and ^{13}C NMR spectra were recorded at room temperature (~25 °C) using a 500 MHz JNM-ECA 500 instrument (JEOL Ltd., Tokyo, Japan) and tetramethylsilane (TMS) as an internal standard. High-resolution mass spectrometry (HRMS) data were recorded using electrospray ionization (ESI) on micrOTOF II (Bruker Daltonics, Billerica, MA, USA) instruments.

2-2. Measurements of the photophysical properties: Absorption and fluorescence spectra were recorded using a V-670 UV–vis spectrophotometer and an FP-8600 spectrofluorometer (both from JASCO Corporation, Tokyo, Japan), respectively, using quartz cells with a path length of 1 cm. Fluorescence quantum yields (Φ) were measured using a C9920-02 instrument (Hamamatsu Photonics K. K., Hamamatsu, Japan) equipped with an integrating sphere, with a measurement error of ±3%. For dyes exhibiting low Φ values (less than 0.01), the values were determined by a relative method using the equation below, which provided better reproducibility.

$$\Phi_{sample} = \Phi_{reference} \times \frac{I_{sample}}{I_{reference}} \times \frac{A_{reference}}{A_{sample}} \times \left(\frac{n_{sample}}{n_{ref}}\right)^2$$

where *I* is the integrated fluorescence intensity, *A* is absorbance at the excitation wavelength, and *n* is the refractive index of the solvent.

2-3. Cellular imaging using confocal laser scanning fluorescence microscopy: Cells were transferred from standard culture dishes to a glass-bottom culture plate (EZVIEW, AGC Techno Glass, Tokyo, Japan) prior to confocal imaging. After allowing for cell adhesion, the medium was removed, and the cells were washed three times with PBS. The cells were then incubated in a mixed solution of MemGlow 488 (0.2 µM) and **dSQ12AQ** (0.2 µM) in Hank's balanced salt solution (HBSS) for 10 minutes at room temperature (approximately 25 °C).

Live-cell imaging was performed using a downlight-type FV1000D laser scanning confocal microscope (Olympus, Tokyo, Japan). Excitation wavelengths were set at 473 nm for MemGlow 488 and 635 nm for **dSQ12AQ**. Emission wavelengths were set at 490–525 nm (green channel, MemGlow 488) and 655–755 nm (red channel, **dSQ12AQ**). Images were acquired using a 60× oil-immersion objective lens and analyzed with the Olympus Fluoview software (ver. 4.2b). Pearson's correlation coefficient (R value) between the green and red channels was calculated using the Coloc 2 macro in Fiji.[5]

To quantitatively evaluate the performance of **dSQ12AQ** in *in vitro* imaging and to compare it with that of **dSQ12S**, we employed an additional advanced confocal microscope, a Nikon Eclipse Ti2

inverted microscope equipped with an AX confocal laser scanning unit and controlled by NIS-Elements software (Nikon Corporation, Tokyo, Japan). The imaging setup for **dSQ12AQ** and **dSQ12S** was as follows: the excitation wavelength was set at 640 nm. And the emission wavelength range was set at 650–720 nm. Images were acquired using a 40×objective lens (Nikon Plan Apo 40x Lambda DIC N2, NA 0.95). All images were processed using the "Denoise AI" functionality in NIS-Elements software.

2-4. *In vivo* imaging using two-photon microscopy (2PM): Approximately 100 μL of **dSQ12AQ** in PBS (10 mg/mL, corresponding to 7.3 mM) was injected into the eyeground vein of a mouse. For bone marrow imaging, the scalp and periosteum above the skull were removed prior to imaging. 3 to 5 hours after injection, the skull bone or hind paw skin was directly imaged using an upright laser-scanning microscope customized for in vivo imaging (A1R MP+, Nikon), equipped with a femtosecond pulsed laser (Insight DS+, 140 fs, 80 MHz; Spectra-Physics) and a 25× water-immersion objective lens (Apo LWD 25× NA 1.10).
The excitation wavelength was set to 1100 nm. All images were acquired using a Galvano scanner at a scanning speed of 0.5 frames per second. Signals from second harmonic generation (SHG) and fluorescence were collected at 500–550 nm and 560–685 nm, respectively, using a GaAsP-type photomultiplier tube. Z-stack images were acquired from the deepest plane to the surface (step size: 1 or 2 μm), covering an area of 512 × 512 μm^2 (1024× 1024 pixels, 0.5 μm/pixel). Reconstructed 3D image stacks were processed using NIS-Elements software with a 3×3 median filter.

***Note on *in vivo* use of membrane probes:**
In our preliminary experiments, intravenous administration of **dSQ12S** or MemGlow 640 prepared either in buffer with insufficient dispersibility or in buffer containing an excessive amount of DMSO to achieve sufficient dispersion resulted in acute adverse effects, including distress and poor survival outcomes. These observations suggest that the insufficient aqueous dispersibility of these probes significantly limits their applicability for intravenous use in live animals. In consideration of animal welfare, we decided not to proceed with further *in vivo* studies using these and excluded these data from this manuscript.

3. Synthesis of dSQ12AQ and Cy5-12AQ

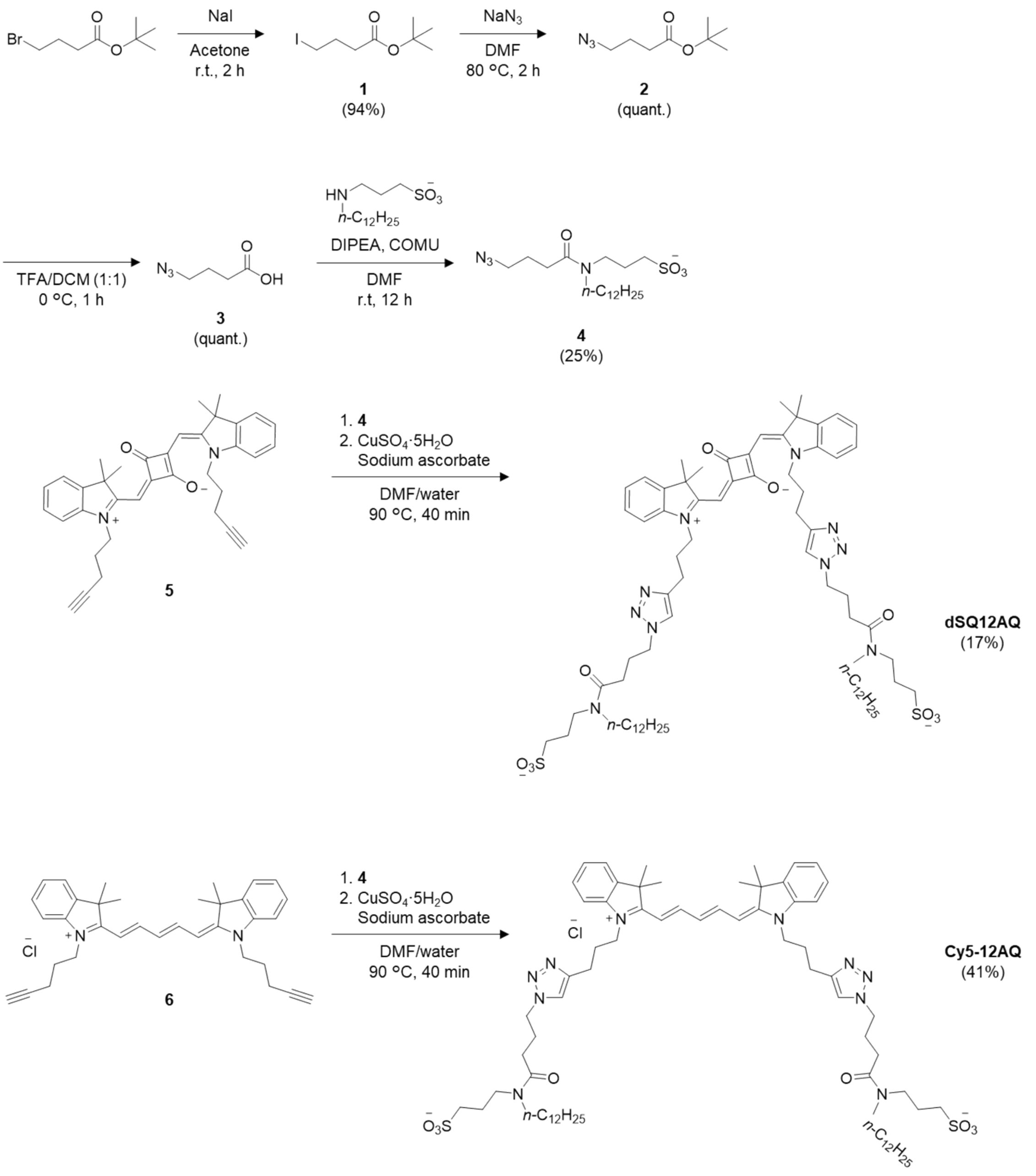

Scheme S1. Synthesis of **dSQ12AQ** and **Cy5-12AQ**.

Synthesis of compound 1

Sodium iodide (6.7 g, 44.8 mmol) was added to a solution of 4-bromobutanoic acid tert-butyl ester (5.0 g, 22.4 mmol) in acetone (56 mL), and the mixture was stirred at room temperature (~25 °C) under an argon atmosphere for 2 h. EtOAc was then added, and the resulting solution was washed successively with water and brine, dried over anhydrous $MgSO_4$, and filtered. The solvents were

removed under reduced pressure to afford compound 1 as a yellowish liquid (5.7 g, 94%). This compound was used for the next reaction without further purification.
^{1}H NMR (500 MHz, $CDCl_3$, ppm): δ = 3.19 (t, 2H, J = 6.8 Hz), 2.32 (t, 2H, J = 7.1 Hz), 2.05 (tt, 2H J = 6.8 Hz, 7.1 Hz), 1.41 (s, 9H,).

Synthesis of compound 2

Sodium azide (0.60 g, 9.3 mmol) was added to a solution of compound **1** (1.0 g, 3.7 mmol) in DMF (12 mL), and the mixture was stirred at 80 °C for 1 h. EtOAc was then added, and the resulting solution was washed successively with water three times, dried over anhydrous Na_2SO_4, and filtered. The solvents were removed under reduced pressure to afford compound **2** as a colorless liquid (677 mg, quant.). This compound was used for the next reaction without further purification.
^{1}H NMR (500 MHz, [D$_6$]DMSO, ppm): δ = 3.30 (t, 2H, J = 6.8 Hz), 2.23 (t, 2H, J = 7.2 Hz) 1.70 (tt, 2H, J = 6.8 Hz, 7.2 Hz), 1.36 (s, 9H).

Synthesis of compound 3

A solution of compound **2** (667 mg, 3.65 mmol) in DCM (7.3 mL) was cooled to 0°C. TFA (7.3 mL) was added, and the mixture was stirred at 0 °C for 1 h. Toluene (20 mL) was then added, and the solvents were removed under reduced pressure to afford compound 3 as a colorless liquid (504 mg, quant.).
^{1}H NMR (500 MHz, [D$_6$]DMSO, ppm): δ = 3.31 (t, 2H, J = 6.8 Hz), 2.25 (t, 2H, J = 7.2 Hz), 1.71 (tt, 2H, J = 6.8 Hz, 7.2 Hz).

Synthesis of compound 4

To a solution of compound **3** (50 mg, 0.39 mmol) in anhydrous DMF (10 mL), COMU (174 mg, 0.41 mmol) and DIPEA (132 µL, 0.77 mmol) were added, and the mixture was stirred at room temperature for 5 min under an argon atmosphere. Separately, 3-(dodecylammonio)propane-1-sulfonate (125 mg, 0.41 mmol) and DIPEA (132 µL, 0.77 mmol) were dissolved in anhydrous DMF (10 mL), and this solution was added to the reaction mixture. The resulting mixture was stirred at room temperature for 12 h. The mixture was filtered, and the solvent was removed under reduced pressure. The residue was dissolved in EtOAc and washed with water, dried over anhydrous $MgSO_4$, and filtered. The solvent was again removed under reduced pressure, and the crude product was purified by flash column chromatography on silica gel (from DCM to DCM/MeOH = 90:10) to afford compound **4** as a brown amorphous solid (41 mg, 25%).
^{1}H NMR (500 MHz, CD_3OD, ppm): δ = 3.52–3.42 (m, 2H), 3.38–3.29 (m, 4H), 2.85–2.71 (m, 2H), 2.54–2.37 (m, 2H), 2.08–1.93 (m, 2H), 1.90–1.77 (m, 2H), 1.65–1.45 (m, 2H), 1.41–1.06 (m, 18H), 0.89 (t, 3H, J = 6.9 Hz).

Synthesis of dSQ12AQ

Sodium L-ascorbate (7.7 mg, 0.039 mmol), copper(II) sulfate pentahydrate (7.0 mg, 0.028 mmol) were dissolved in water (300 µL) in a test tube and stirred until the solution turned orange (ca. a few minutes). This aqueous solution was then added to a solution of compound **4** (52 mg, 0.13 mmol) and compound **5** (30 mg, 0.057 mmol) in DMF (1 mL). The resulting mixture was stirred at 90 °C for 40 min. The solvent was removed under reduced pressure, and the residue was purified by column chromatography on C_{18}-reversed phase silica gel (from H_2O/MeOH = 1:1 to 1:3) to afford final compound **dSQ12AQ** as a blue solid (14 mg, 17%).

It should be noted that the ^{1}H NMR spectrum of this molecule could not be fully assigned due to its highly complex structure, including a mixture of rotamers resulting from restricted rotation around two tertiary amide bonds. However, the integration ratios of characteristic signals corresponding to squaraine aromatic protons, triazole ring, and α-methylene protons were consistent with the theoretical values. Furthermore, HRMS confirmed the presence of the target compound. Based on these results, we concluded that the desired molecule, **dSQ12AQ** was successfully synthesized.

^{1}H NMR (500 MHz, CD_3OD, ppm): δ = 7.95–7.79 (m, 2H), 7.50–7.38 (m, 2H), 7.38–7.26 (m, 2H), 7.25–7.09 (m, 4H), 6.00–5.88, 4.55–4.34, (m, 4H), 4.30–4.02, (m, 4H), 3.56–3.33, (m, 4H), 3.27–3.09 (m, 4H), 2.95–2.85 (m, 4H), 2.85–2.69 (m, 4H), 2.51–2.40 (m, 4H), 2.40–2.31 (m, 4H), 2.26–2.07 (m, 8H), 2.06–1.90 (m, 4H), 1.73 (s, 12H), 1.56–1.41 (m, 4H), 1.38–1.15 (m, 36H), 0.93–0.76.

HRMS (ESI): *m/z*: calcd for $C_{74}H_{110}N_{10}O_{10}S_2^{2-}$: 681.3929 $[M]^{2-}$; found: 681.3898.

Synthesis of Cy5-12AQ

Sodium L-ascorbate (9.0 mg, 0.045 mmol) and copper(II) sulfate pentahydrate (8.0 mg, 0.032 mmol) were dissolved in water (375 µL) in a test tube, and stirred until the color of the solution turned orange (ca. a few minutes). This aqueous solution was then added to a solution of compound **4** (59 mg, 0.14 mmol) and compound **6** (38 mg, 0.064 mmol) in DMF (1.3 mL). The resulting mixture was stirred at 90 °C for 1 h. The solvent was removed under reduced pressure, and the residue was dissolved in a 1:1 mixture of DCM and MeOH, then filtered through Celite. The solvents were again removed under reduced pressure, and the crude product was purified by size-exclusion column chromatography on Sephadex LH-20 (pre-swollen in DCM/MeOH = 1:1) to afford a target compound, **Cy5-12AQ**, as a blue solid (40 mg, 41%). Characterization of this compound was performed in the same manner as described for dSQ12AQ.

^{1}H NMR (500 MHz, CD_3OD, ppm): δ = 8.23–7.83 (m, 4H), 7.49–7.40 (m, 2H), 7.39–7.32 (m, 2H), 7.31–7.14 (m, 4H), 6.61 (br, 1H), 6.46–6.08 (m, 2H), 4.55–4.35 (m, 4H), 4.28–4.03 (m, 2H), 3.49–3.35 (m, 4H), 3.23–3.15 (m, 2H), 3.02–2.81 (m, 4H), 2.80–2.70 (m, 4H), 2.49–2.28 (m, 4H), 2.27–

2.05 (m, 4H), 2.03–1.89 (m, 4H), 1.69 (s, 12H), 1.56–1.40 (m, 4H), 1.31–1.13 (m, 38H), 0.91–0.79 (m, 6H).

HRMS (ESI): *m/z*: calcd for $C_{73}H_{113}N_{10}O_8S_2^-$: 1321.8190 [*M*]$^-$; found: 1321.8164.

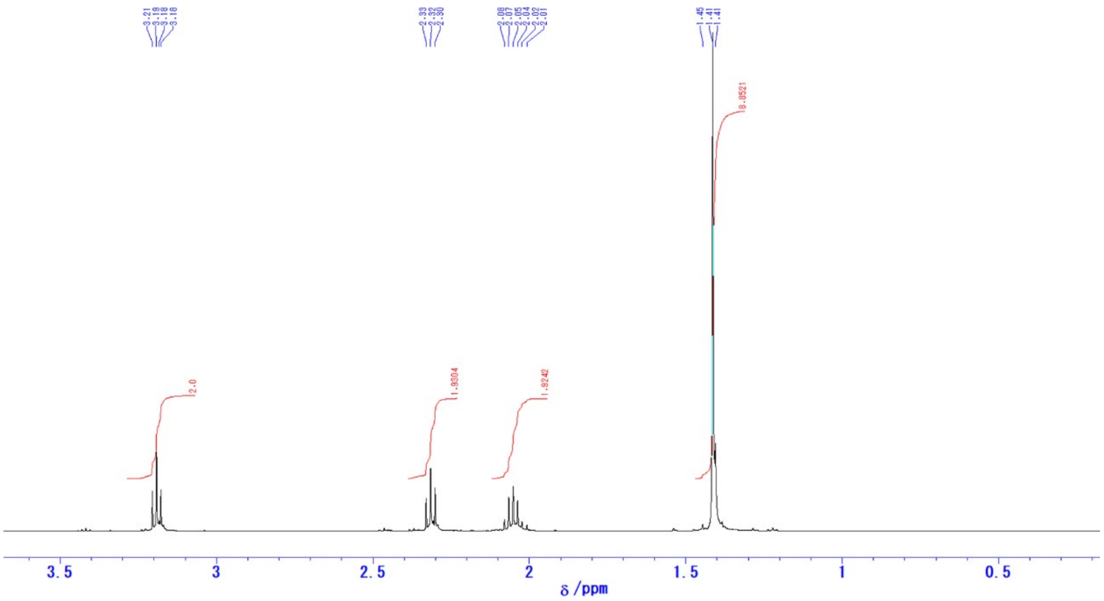

Figure S1. ^{1}H NMR spectrum of compound **1** ($CDCl_3$).

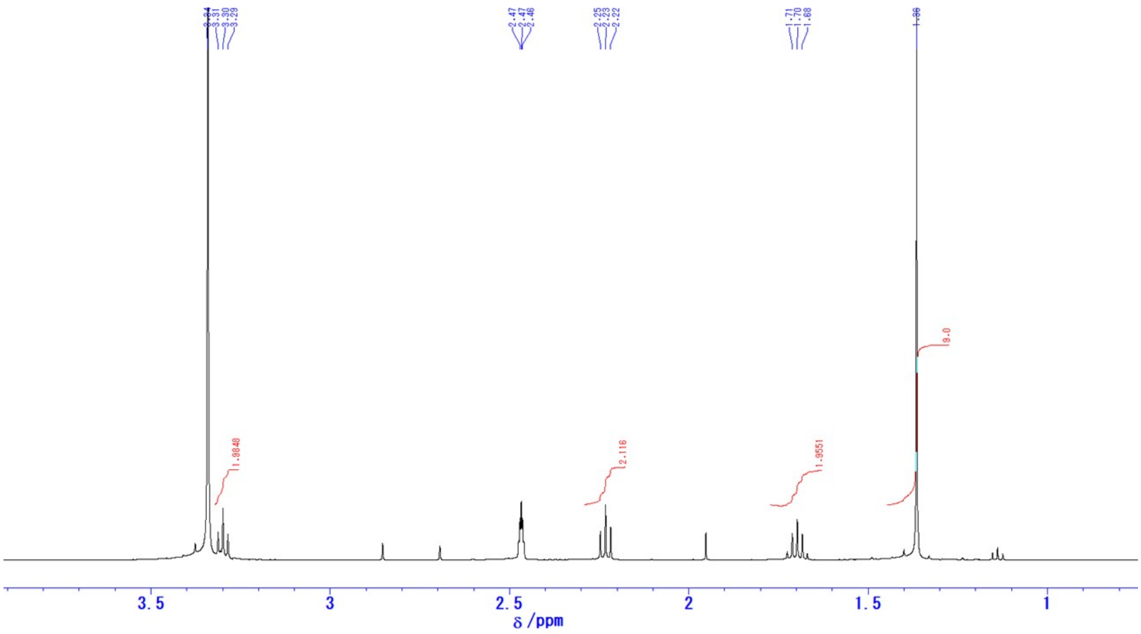

Figure S2. ^{1}H NMR spectrum of compound **2** ([D_6]DMSO).

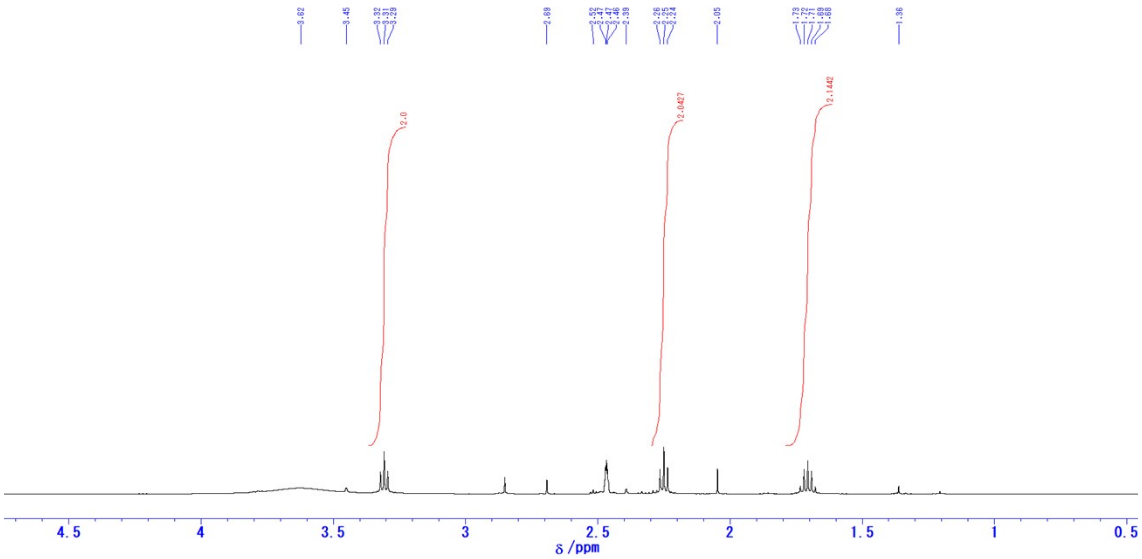

Figure S3. ^{1}H NMR spectrum of compound **3** ([D$_6$]DMSO).

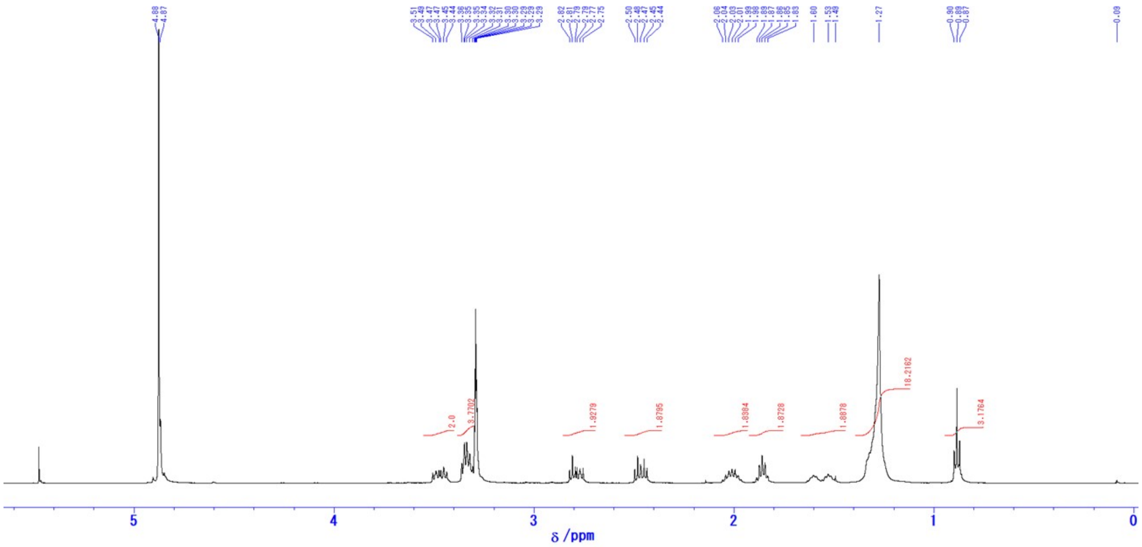

Figure S4. ^{1}H NMR spectrum of compound **4** (CD$_3$OD).

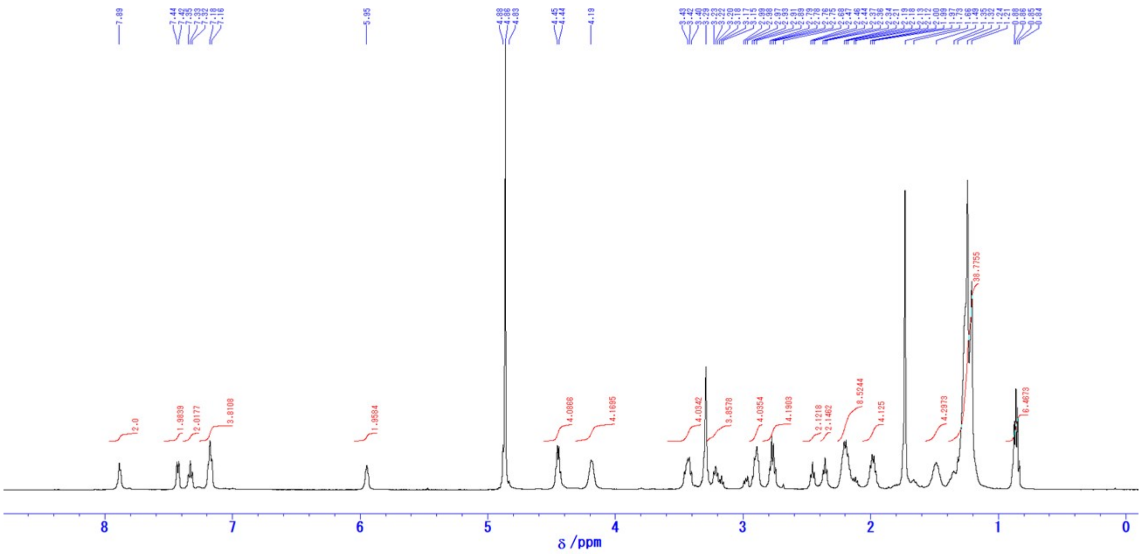

Figure S5. ^{1}H NMR spectrum of compound **dSQ12AQ** (CD_3OD).

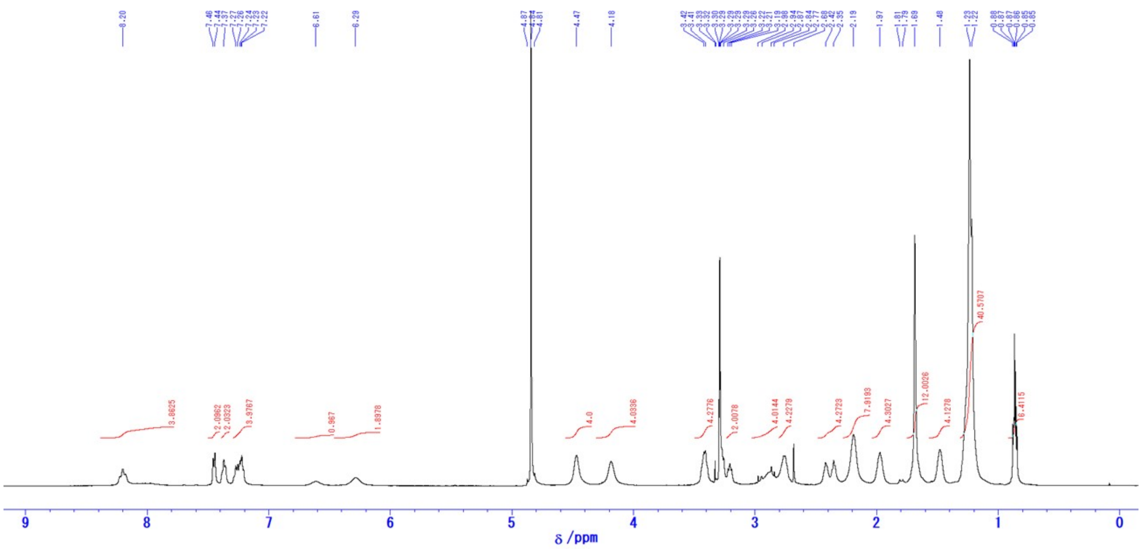

Figure S6. ^{1}H NMR spectrum of compound **Cy5-12AQ** (CD_3OD).

4. Aggregation behavior of dSQ12AQ and dSQ12S in PB

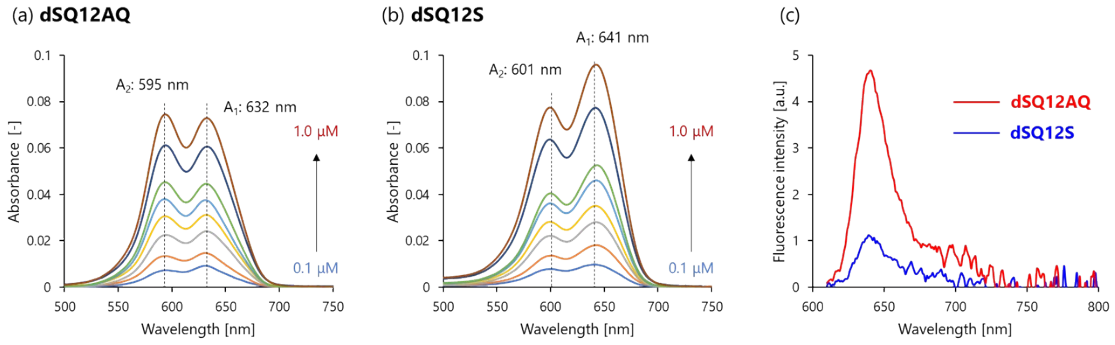

Figure S7 Dependence of the absorption spectral shape of (a) **dSQ12AQ** and (b) **dSQ12S** in PB on dye concentration. (c) Fluorescence spectra of **dSQ12AQ** and **dSQ12S** in PB. The probe concentration was 0.1 µM. Excitation was performed at 600 nm, at which the absorbance of **dSQ12AQ** and **dSQ12S** was 0.0071 and 0.0078, respectively.

Determination of critical aggregation concentration (CAC)

The CAC of **dSQ12AQ** was estimated by plotting the ratio of absorbance at 632 nm and 595 nm (A_1/A_2), obtained from Figure S6, as a function of dye concentration. The A_1/A_2 ratio exhibited a sharp increase at low concentrations, followed by a plateau at higher concentrations (see Figure S8 below), which is characteristic of aggregate formation. The data were fitted using a piecewise linear model comprising two linear segments, and the intersection point was taken as the CAC. The CAC of **dSQ12AQ** was determined to be approximately 0.42 µM. In contrast, **dSQ12S** showed no appreciable change in the absorbance ratio at 641 nm and 601 nm (A_1/A_2) across the examined concentration range. Linear regression analysis indicated no statistically significant concentration dependence ($p = 0.32$), suggesting that the CAC of **dSQ12S** lies outside the tested concentration.

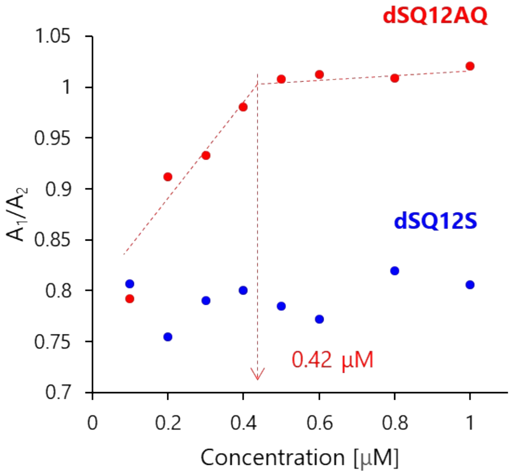

Figure S8. (a) Determination of the CAC of **dSQ12AQ** (the detailed method is described above).

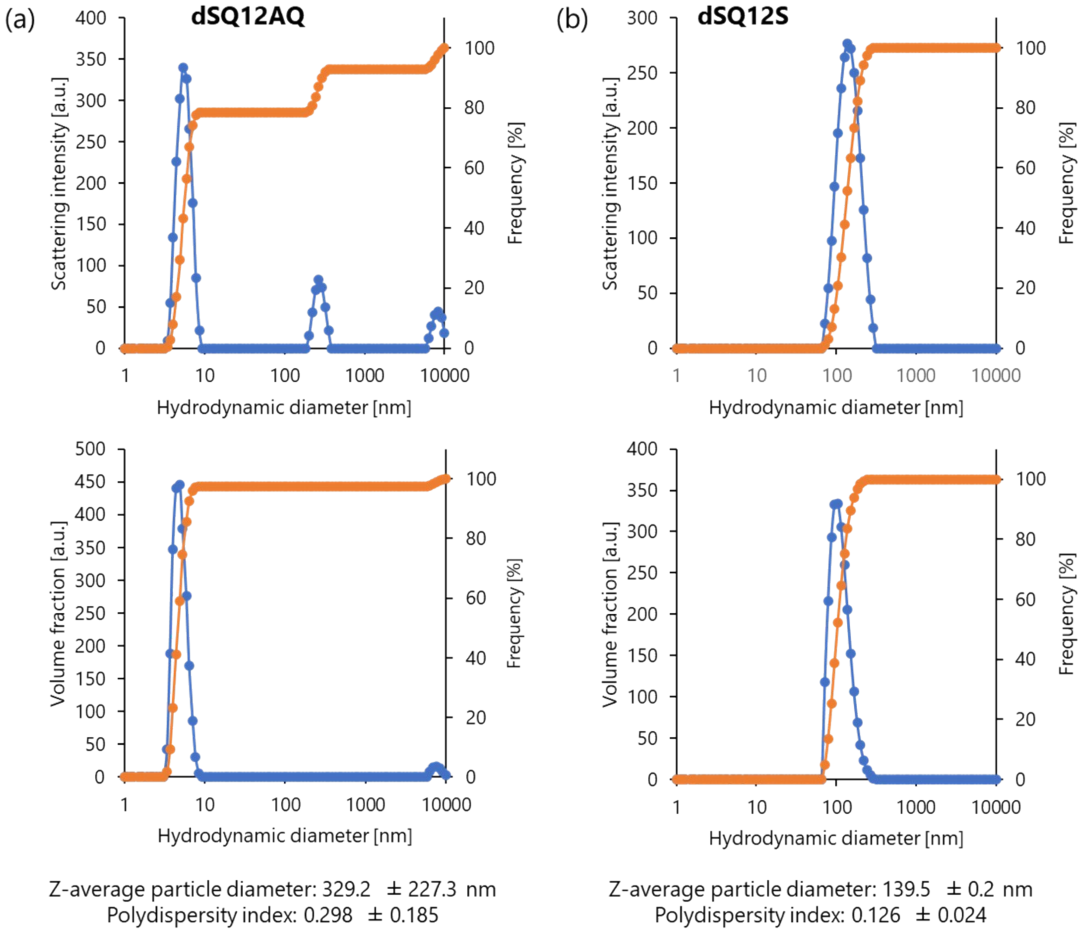

Figure S9. Intensity-weighted particle size distribution (top, y-axis: scattering intensity) and volume-weighted particle size distribution (bottom, y-axis: volume fraction [a.u.]) of (a) **dSQ12AQ** and (b) **dSQ12S** in PB**,** measured by DLS. The **dSQ12S** solution containing 1% DMSO to maintain a dispersed state. The plots show intensity/fraction (left axis) and frequency (right axis) as a function of hydrodynamic diameter. The cumulant-derived Z-average particle diameters and polydispersity indices were determined from three independent measurements. Note that the DLS system use here was ELSZ 2000ZS (Otsuka Electronics Co. Ltd., Osaka, Japan) equipped with a 783 nm laser, as the dyes exhibit absorption and fluorescence at around 640 nm, which is incompatible with typical DLS instruments operating at similar wavelengths.

5. Spectroscopic properties of dSQ12AQ and dSQ12S

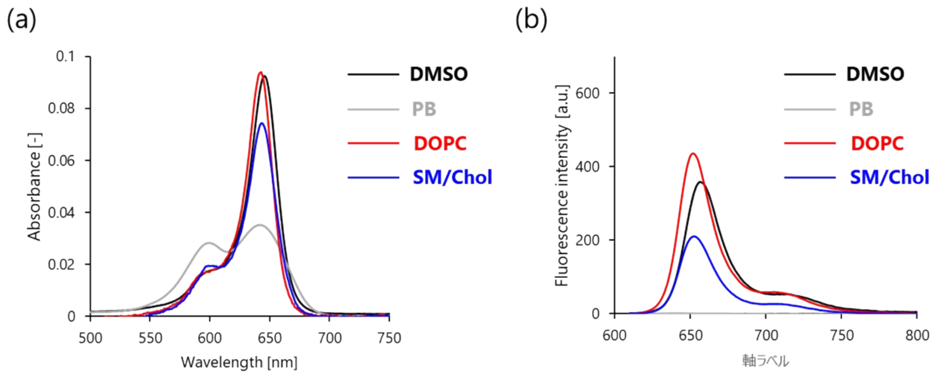

Figure S10. (a) Absorption and (b) fluorescence spectra of **dSQ12S** in DMSO, PB, and in the presence of LUVs. The probe and lipid concentrations were 0.4 and 80 μM, respectively (lipid only for LUVs).

Table S1. Spectroscopic parameters of **dSQ12AQ** and **dSQ12S** in different environments. λ_{abs}, absorption maximum wavelength; ε, molar extinction coefficient; λ_{em}, fluorescence maximum wavelength; Φ, fluorescence quantum yield. For PB, two distinct absorption maxima were observed, and both λ_{abs} and ε values are reported.

	dSQ12AQ				dSQ12S			
	λ_{abs} [nm]	ε [10^5 M^{-1} cm^{-1}]	λ_{em} [nm]	Φ	λ_{abs} [nm]	ε [10^5 M^{-1} cm^{-1}]	λ_{em} [nm]	Φ
DMSO	645	2.1	656	0.29	646	2.1	658	0.31
PB (0.1 μM dye)	632, 595	0.93, 0.74	642	0.008[a]	640, 601	0.97, 0.78	643	0.002[b]
PB (0.5 μM dye)	632, 593	0.75, 0.76	642	0.004[a]	642, 600	0.92, 0.72	644	< 0.001
DOPC	642	2.3	652	0.46	642	2.3	652	0.39
SM/Chol	643	2.0	653	0.30	643	0.19	652	0.21

a: Determined by relative method, where Φ value of **dSQ12AQ** in the presence of DOPC LUVs was used as a reference.

b: Determined by relative method, where Φ value of **dSQ12S** in the presence of DOPC LUVs was used as a reference.

6. *In vitro* imaging comparison between dSQ12AQ and dSQ12S

Calculation of signal-to-noise ratio (SNR) for confocal imaging of cell membranes.

The SNR of the confocal membrane images was calculated as follows:

1. Images were acquired under fixed imaging conditions (laser power, detector gain, and other parameters were constant).
2. For each field of view, the background intensity and its standard deviation were measured from three randomly selected regions outside the cells. The mean background intensity (B) and the average standard deviation (σ) of these three regions were used for the SNR calculation.
3. For each cell, the plasma membrane was manually outlined using the Freehand line tool (3 px width) in Fiji. Three independent measurements were performed per cell (using slightly different line positions along the membrane), and the mean membrane signal intensity (S) was obtained by averaging the three measurements.
4. SNR was calculated as:

   $$SNR = {}^{(S-B)}/_{\sigma}$$

5. A total of ~10 cells from multiple fields of view (z-position kept as consistent as possible) were analyzed, and the average SNR ± standard deviation was reported.

As noted in the main text, we determined the SNR of **dSQ12AQ** and **dSQ12S** to be 142 ± 38 (mean ± SD) and 212 ± 41, respectively.

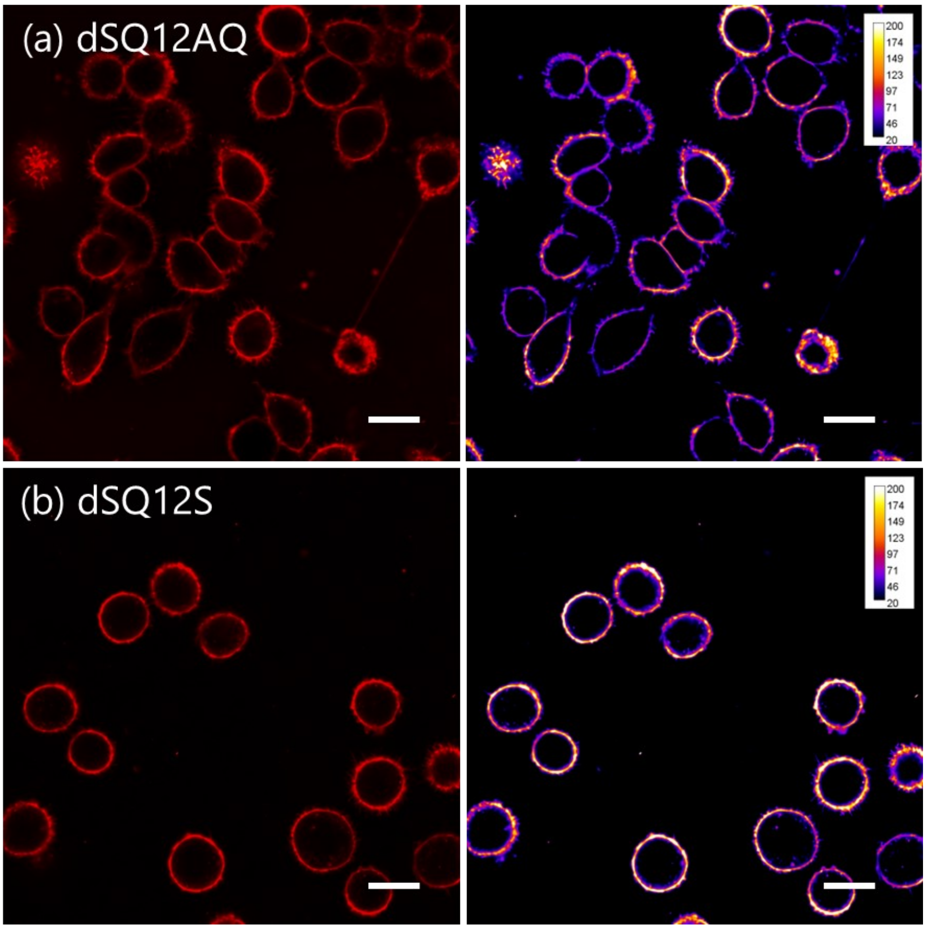

Figure S11. Laser scanning confocal microscopy images of unwashed PC-3 cells stained with (a, left) 10 nM (a) **dSQ12AQ** and (b) **dSQ12S**. Left: original fluorescence image (Ex: 640 nm, Em: 650-720 nm), right: heatmap of SNR. Imaging conditions: laser power, 20.0%; detector gain, 18.0%; pinhole size 0.7 AU; frame averaging, 1; excitation dwell time, 0.505 frames/s; pixel size. 0.173 μm; frame size: 177 × 177 μm^2 (1024× 1024 pixels, 0.173 μm/pixel). Scale bar is 20 μm.

7. *In vivo* 2PM imaging using Cy5-12AQ

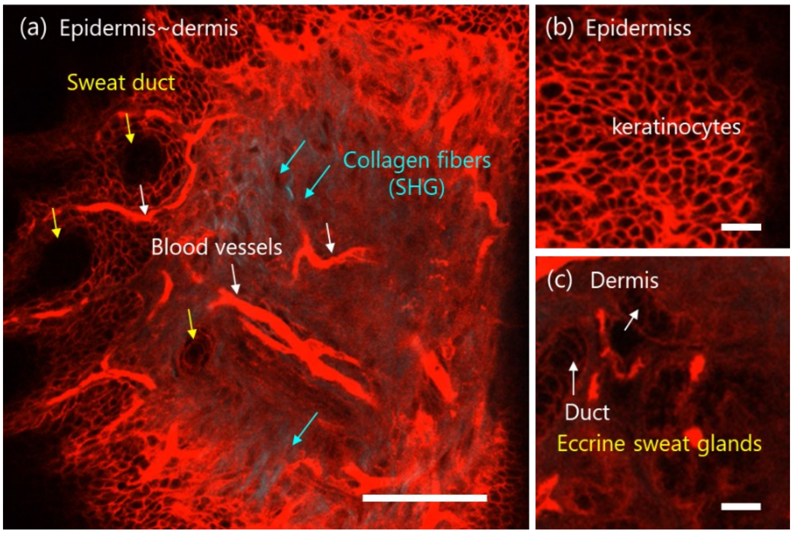

Figure S12. 2PM images of the skin tissue of a living mouse, acquired 5 hours post intravenous injection of **Cy5-12AQ** (100 µL, 10 mg/mL). (a) Overview of the tissue structure covering from the epidermis to the dermis. Image of (b) keratinocytes in the epidermis. (d) Image of the eccrine sweat glands in the dermis region. Ex: 1100 nm, Em: 500-550 nm (cyan, SHG), 560-685 nm (red, fluorescence).

8. Supplemental Movies

Movie S1. Time-lapse imaging of the skull bone marrow in a living mouse.

Movie S2. Time-lapse imaging of a blood vessel within the skull bone marrow.

Movie S3. Three-dimensional imaging of the hind paw skin tissue in a living mouse.

9. References

1. M. Collot *et al.*, *Chem. Commun.*, 2015, **51**, 17136–17139.
2. M. Collot *et al.*, *Cell Chem. Biol.*, 2019, **26**, 600–614.e7.
3. D. I. Danylchuk, S. Moon, K. Xu and A. S. Klymchenko, *Angew. Chem. Int. Ed.*, 2019, **58**, 14920–14924.
4. M. J. Hope, M. B. Bally, G. Webb, P. R. Cullis, *Biochim. Biophys. Acta.*, 1985, **812**, 55–65.
5. J. Schindelin, I. Arganda-Carreras, E. Frise, V. Kaynig, M. Longair, T. Pietzsch, S. Preibisch, C. Rueden, S. Saalfeld, B. Schmid, J.-Y. Tinevez, D. J. White, V. Hartenstein, K. Eliceiri, P. Tomancak, A. Cardona, *Nat Methods*, 2012, **9**, 676–682.
